# TGF-β–independent CTGF induction regulates cell adhesion mediated drug resistance by increasing collagen I in HCC

**DOI:** 10.18632/oncotarget.15521

**Published:** 2017-02-20

**Authors:** Yeonhwa Song, Jin-Sun Kim, Eun Kyung Choi, Joon Kim, Kang Mo Kim, Haeng Ran Seo

**Affiliations:** ^1^ Cancer Biology Research Laboratory, Institut Pasteur Korea, Bundang-Gu, Seongnam-Si, Gyeonggi-Do, 13488, Korea; ^2^ Division of Gastroenterology and Hepatology, ASAN Medical Center, Seoul, 05505, Korea; ^3^ Division of Radiation Oncology, ASAN Medical Center, Seoul, 05505, Korea; ^4^ Laboratory of Biochemistry, Division of Life Sciences, Korea University, Seoul, 02841, Korea

**Keywords:** tumor spheroids, collagen 1A1, connective tissue growth factor (CTGF), cell adhesion-mediated drug resistance (CAM-DR), hepatocellular carcinoma

## Abstract

Hepatocellular carcinoma (HCC) is resistant to conventional chemotherapeutic agents and remains an unmet medical need. Here, we demonstrate a mechanism of cell adhesion-mediated drug resistance using a variety of HCC spheroid models to overcome environment-mediated drug resistance in HCC. We classified spheroids into two groups, tightly compacted and loosely compacted aggregates, based on investigation of dynamics of spheroid formation. Our results show that compactness of HCC spheroids correlated with fibroblast-like characteristics, collagen 1A1 (COL1A1) content, and capacity for chemoresistance. We also showed that ablation of COL1A1 attenuated not only the capacity for compact-spheroid formation, but also chemoresistance. Generally, connective tissue growth factor (CTGF) acts downstream of transforming growth factor (TGF)-β and promotes collagen I fiber deposition in the tumor microenvironment. Importantly, we found that TGF-β–independent CTGF is upregulated and regulates cell adhesion-mediated drug resistance by inducing COL1A1 in tightly compacted HCC spheroids. Furthermore, losartan, which inhibits collagen I synthesis, impaired the compactness of spheroids via disruption of cell-cell contacts and increased the efficacy of anticancer therapeutics in HCC cell line- and HCC patient-derived tumor spheroids. These results strongly suggest functional roles for CTGF-induced collagen I expression in formation of compact spheroids and in evading anticancer therapies in HCC, and suggest that losartan, administered in combination with conventional chemotherapy, might be an effective treatment for liver cancer.

## INTRODUCTION

Hepatocellular carcinoma (HCC) is the sixth most common malignant tumor type and the second leading cause of cancer-related deaths in the world [1]. The highest incidence occurs in Eastern Asia and sub-Saharan Africa. The prognosis for this disease is very poor. Only 10%–20% of liver tumors can be removed surgically, and the recurrence rate is approximately 80% after resection, resulting in a high rate of mortality [2].

Importantly, most HCCs are resistant to conventional chemotherapeutic agents [3]. Researchers have attempted to identify novel target genes and drug candidates for HCC; however, development of targeted drugs has not yet significantly improved outcomes.

In view of all this, the concept of cancer biology is changing from a focus on the genetics of tumor cells alone to also exploring the complex interplay between cancer cells and the tumor microenvironment (TME). The TME is the extracellular environment in which the tumor exists, including surrounding blood vessels, immune cells, fibroblasts and other cells, signaling molecules, and the extracellular matrix (ECM) [4–6]. Environment-mediated drug resistance can be largely subdivided into two categories, soluble factor-mediated drug resistance (SFM-DR) and cell adhesion-mediated drug resistance (CAM-DR) [7].

In this study, we formed various HCC spheroids to study CAM-DR, which is mediated by cell-cell and cell-ECM interactions, and determined the relationship between the capacity for spheroid formation and chemoresistance.

To date, monolayer culture-based assay models have dominated cancer biology and preclinical cancer drug discovery efforts. However, these models fail to predict *in vivo* efficacy, contributing to a lower success rate for translating a new investigational drug to clinical approval. Recently, scientists have highlighted the need for complex 3D cell-culture systems in oncology research because tumor spheroids strikingly mirror the 3D *in vivo* context, as well as therapeutically relevant pathophysiological gradients of *in vivo* tumors such as pH, oxygen, nutrient, and drug concentrations [8].

ECM proteins in the TME play important roles in liver function in health and disease. Abnormal ECM composition and structure in solid tumors are the major obstacles for penetration of anticancer drugs. Collagens are the most abundant structural ECM protein in liver. Disproportionate levels of collagens result in altered cellular phenotypes and architectural distortion with abnormal blood flow. Moreover, among ECM proteins, elevated collagen level is a key barrier to interstitial drug penetration and, thereby, reduces the efficacy of chemotherapeutics [9, 10].

CAM-DR is facilitated by signaling cascades initiated by growth factors present in the TME. In particular, connective tissue growth factor (CTGF, also known as CCN2) is preferentially produced by tumor cells, and elevated CTGF expression in tumor cells significantly correlates with poor clinical prognosis. Because CTGF is overexpressed in fibrotic human liver, it has been recognized as a key profibrogenic factor in that organ [11]. CTGF has been isolated from other important molecular targets in HCC, attesting to the potential relevance of CTGF in HCC progression. Recently, CTGF expression was reported to be elevated in HCC tissues, and HCC patients with high serum CTGF levels show reduced survival, supporting the potential relevance of CTGF in HCC progression [12, 13]. In this study, we demonstrated that high CTGF expression contributes to compact-spheroid formation through elevation of COL1A1 and enables evasion of anticancer therapies in HCC spheroids.

## RESULTS

### Capacity for compacted spheroid formation is consistent with sorafenib sensitivity

**Figure 1:**
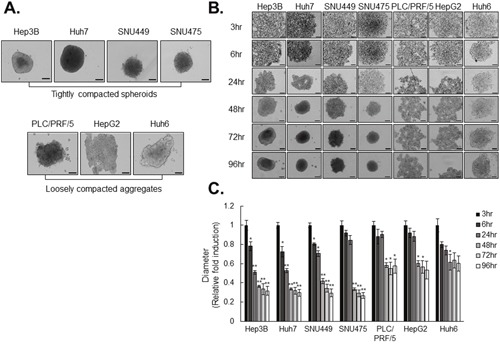
HCC cell lines differ in spheroid-forming capacity **A**. Spheroid was formed for 3 days using HCC cell lines. **B**. HCC cell lines were cultured in 96-well ultra-low attachment (ULA) plate for 96hr. Images of spheroids were obtained at indicated times. **C**. Kinetic of spheroids formation was measured in bright-field images using Operetta analysis software. The Values were normalized to 3hr of each cell lines. Data represent the mean values ± SD from three independent experiments. All bright-field images of spheroids were obtained using the Operetta® High Content Screening System and a 10× objective. *P<0.05, **P<0.005. Scale bar = 200μm.

We determined the dynamics of HCC cell line-derived spheroid formation using seven HCC cell lines to investigate the relationship between capacity of spheroid formation and sorafenib resistance. For analysis of spheroid stiffness, we measured the size and observed the morphology of each spheroid. Among the seven spheroid types, Hep3B, Huh7, SNU449, and SNU475 cells formed relatively tightly compacted spheroids, whereas PLC/PRF/5, Huh6, and HepG2 cells exhibited loosely compacted aggregates with less tightly associated cells without cell death (Figure 1A, Supplementary Figure 1, and 2). Kinetics of the rate of spheroid assembly showed that Hep3B, Huh7, SNU449, and SNU475 (tightly compacted spheroid-forming cell lines) displayed strong cohesion in short periods of time, whereas PLC/PRF/5, Huh6, and HepG2 cells (loosely compacted aggregate-forming cell lines) displayed limited ability to enhance spheroid stiffness (Figure 1B, 1C).

**Figure 2:**
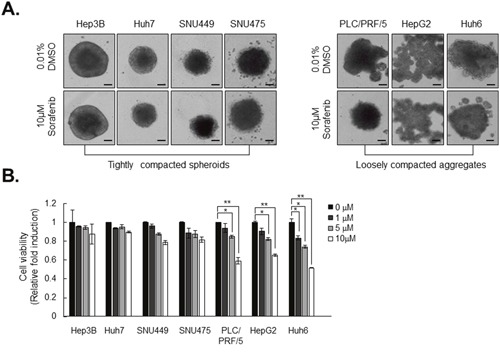
Tightly compacted spheroids show strong resistance to sorafenib compared to loosely compacted aggregates **A**. HCC spheroids were formed for 3 days and treated with 10 μM of sorafenib for another 4 days. **B**. Cell viability was measured with resazurin assay. After 5 hr of treatment with 50 μM resazurin, absorbance was measured using an Enspire plate reader. The values were normalized to control (0.01% DMSO). Data represent the mean values ± SD from three independent experiments. All bright-field images of spheroids were obtained at 7 days using the same method. *P<0.05, **P<0.005. Scale bar = 200μm.

To investigate CAM-DR, we determined the relationship between the capacity for spheroid formation and sorafenib resistance. To determine a correlation between compactness of spheroids and sorafenib resistance, the cytotoxic effect of sorafenib was analyzed in the seven types of HCC spheroid. Interestingly, each spheroid treated with 10 uM sorafenib displayed a different cytotoxic effect. Most loosely compacted aggregates displayed significant reduction in size in response to sorafenib, whereas the form of tightly compacted spheroids remained intact (Figure 2A).

This unexpected different sensitivity of HCC spheroids to sorafenib was further confirmed by resazurin assay of cell viability. Treatment with sorafenib significantly decreased cell viability in loosely compacted aggregates in a dose-dependent manner. On the other hand, tightly compacted spheroids exhibited vigorous sorafenib resistance relative to loosely compacted aggregates (Figure 2B). Taken together, these results suggest that resistance to sorafenib is consistent with strong cohesion between the cancer cells in HCC spheroids.

### Compactness of spheroids correlates with fibroblast-like characteristics in HCC cell lines

**Figure 3:**
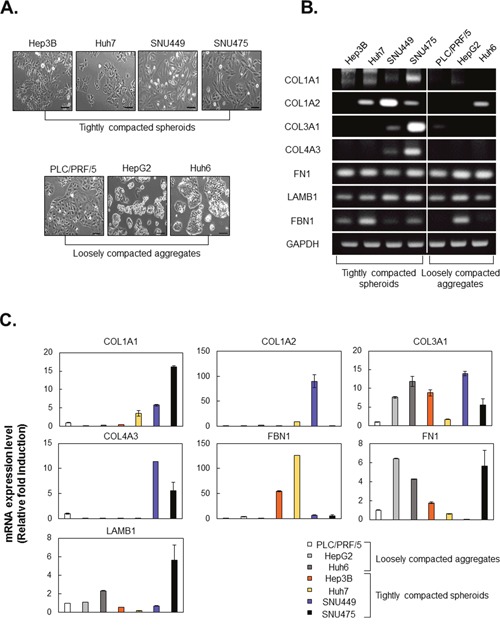
HCC cell lines, which form tightly compacted spheroids, show fibroblast-like morphology and express high levels of ECM-related molecules **A**. Morphology of HCC cell lines in monolayer culture. Bright-field images were obtained using the same method with a 100× objective. **B, C**. Expression of mRNA encoding ECM-related molecules in seven HCC cell lines was evaluated by RT-PCR (B) and real-time PCR (C). GAPDH served as a loading control, and values were normalized to GAPDH. Values were normalized to GAPDH. In real-time PCR, values of PLC/PRF/5 were normalized to 1, and other experiment groups were normalized to PLC/PRF/5. Data are shown as means ± SD from two independent experiments with duplicates. Scale bar = 50μm.

Because compactness of spheroids is related to sorafenib resistance, we investigated factors that might be responsible for differences in spheroid compactness. First, we compared the morphology of tightly compacted spheroid-forming cell lines and loosely compacted aggregate-forming cell lines in monolayer culture and found that the morphology of the former was markedly more fibroblast-like than that of the latter (Figure 3A).

Generally, the ECM occupies a small percentage of the volume of the normal liver, whereas many types of HCC tissue show excessive ECM production or limited ECM turnover. We examined mRNA expression of ECM proteins in tightly compacted and loosely compacted aggreagates. We found that the tightly compacted spheroids expressed higher levels of fibrillar collagens, collagen I and III, and collagen IV. In particular, elevated expression of collagen 1A1 (COL1A1) was observed in all tightly compacted spheroids, whereas loosely compacted aggregates showed expressed very low levels of COL1A1. The pattern of expression of noncollagenous ECM proteins, such as fibronectin, laminins, and fibrillin, did not serve as predictors of spheroid compactness (Figure 3B, 3C).

**Figure 4:**
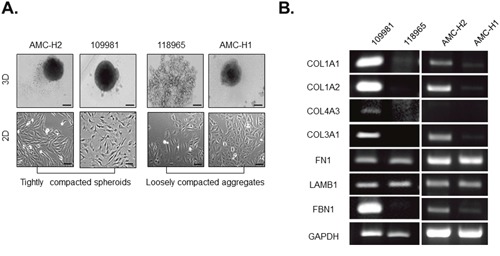
Collagen I expression and fibroblast-like morphology are correlated with compactness of spheroids generated by patient-derived primary HCCs **A**. Spheroid formation and 2D morphology of patient-derived primary HCCs. Spheroids were formed for 3 days. Bright-field images were obtained using the same method with 10× objective for spheroids and 100× objective for monolayer cells. **B**. Expression of mRNA encoding ECM-related molecules in patient-derived primary HCCs was evaluated by RT-PCR. GAPDH served as a loading control. Data represent the mean values ± SD from two independent experiments. Scale bar = 200μm (spheroid), 50μm (monolayer).

We then estimated capacity of the more physiologically relevant primary HCCs for compact-spheroid formation. Similar to the HCC cell lines, primary HCCs were also largely divided into two groups dependent on their capacity for spheroid formation. 109981 and AMC-H2 cells showed strong spheroid compactness, whereas 118965 and AMC-H1 cells did not form rigid spheroids (Figure 4A). Even though AMC-H1 aggregates appeared solid, they could be easily dissociated by mechanical force [data not shown].

In patient-derived primary HCCs, spheroid compactness also correlated with fibroblast-like cell morphology (Figure 4A). Fibrillar collagens were also expressed at a higher level in tightly compacted spheroids produced by patient-derived HCCs than in the corresponding loosely compacted aggregates, similar to the observations for HCC cell lines (Figure 4B).

Thus, these results suggest that fibroblast-like morphology and high collagen expression level are coincident with tightly compacted spheroid formation in HCC.

### Inhibition of COL1A1 expression induces structural change and improves efficacy of chemotherapeutic drugs in HCC spheroids

**Figure 5:**
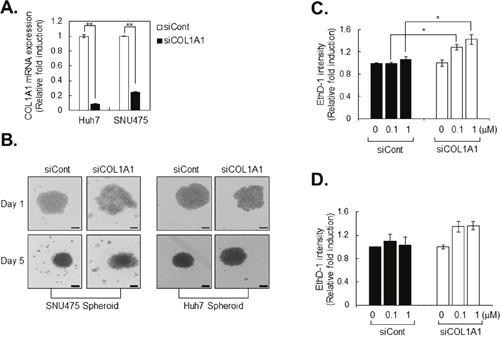
Drug sensitivity is increased in COL1A1 knockdown HCC cell lines **A**. Collagen 1A1 mRNA expression in control (siCont) and COL1A1 knockdown HCC cell lines (siCOL1A1). Expression of mRNA was evaluated by real-time PCR, and the values were normalized to GAPDH. The values of siCOL1A1 were normalized to siCont. **B**. After transfection of HCC cell lines with siCont and siCOL1A1, the capacity of spheroid formation was evaluated for 5 days. **C, D**. After 3 days of spheroid formation, Huh7 spheroids were treated with sorafenib (C) or cisplatin (D) for another 4 days. On the final day of treatment (at 7days from cell seeding), spheroids were stained with ethidium homodimer-1 (EthD-1) to evaluate the extent of cell death. After image acquisition, EthD-1 intensity was measured using the same method. The values were normalized to control (0μM) of each group. Data represent the mean values ± SD from two independent experiments. *p<0.05, **p<0.005. Scale bar = 200μm.

Because high COL1A1 expression was observed in all tightly compacted spheroids, we used siRNA-mediated knockdown of COL1A1. Using control and COL1A1-deficient HCC cells, we compared the morphology of HCC spheroids to investigate the effect of COL1A1-depletion on spheroid compactness. Transfection of siRNA targeting COL1A1 (siCOL1A1) efficiently inhibited COL1A1 mRNA level in SUN475 and Huh7 cells, whereas control siRNA (siCont) treatment did not (Figure 5A). We also investigated the spheroid formation capacity in other collagen-related siRNA (siCOL1A2, siCOL3A1, and siCOL4A3) transfected SNU475 and Huh7 cells, however, they don't show severe morphological change compared to spheroid of COL1A1-deficient cells (Supplementary Figure 3). As expected, COL1A1-deficient SNU 475 and Huh7 cells, which normally express high levels of COL1A1 mRNA, did not form tightly compacted spheroids (Figure 5B). Morphological changes in HCC spheroids induced by depletion of COL1A1 were associated with greater therapeutic efficacy of the anticancer drugs sorafenib and cisplatin, compared with spheroids produced by cells transfected with control siRNA (Figure 5C-5D). Collectively, these results suggest that COL1A1 plays a pivotal role in formation of rigid tumor environments and thereby reduces the efficacy of chemotherapeutics in HCC spheroids.

### CTGF is overexpressed and regulates COL1A1 level in tightly compacted HCC spheroids

Next, we asked which growth factors might facilitate tight cell adhesion by synthesis of COL1A1 in tumor spheroids. After preparing lysates of HCCs cultured under 2D and 3D conditions, we determined expression levels of TGFβ1 and CTGF by western analysis.

**Figure 6:**
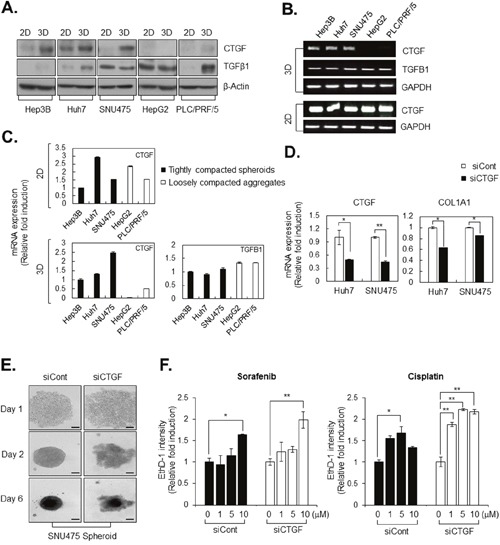
CTGF is overexpressed and regulates COL1A1 level in tightly compacted HCC spheroids **A**. Lysates of HCC cell lines cultured under 2D and 3D conditions were analyzed by western blotting with anti-CTGF, anti–TGFΔ-1, and anti–Δ-actin (control) antibodies. **B, C**. mRNA expression in HCC cell lines cultured under 2D and 3D conditions was evaluated by RT-PCR (B) and real-time PCR (C). CTGF and TGFΔ-1 mRNA levels were examined, and GAPDH was served as a loading control. The values of Hep3B were normalized to 1, and other experiment groups were normalized to Hep3B. **D**. COL1A1 and CTGF mRNA levels in control siRNA transfected (siCont) and CTGF knockdown HCC cell lines (siCTGF) were examined by real-time PCR. In real-time PCR, all values were normalized to GAPDH, and the values of siCTGF were normalized to siCont. **E**. Spheroid formation capacity was observed in SNU475-siCont and SNU475-siCTGF for 6 days. Bright-field images were obtained using the same method. **F**. Drug sensitivity was measured by EthD-1 (dead cell) intensity induced by sorafenib and cisplatin at indicated concentration in Huh7-siCont and Huh7-siCTGF after 4 days from drug treatment. The values were normalized to control (0μM) of each group. Data represent the mean values ± SD from three independent experiments. *P<0.05, **p<0.005. Scale bar = 200μm.

Interestingly, CTGF expression was elevated in tightly compacted HCC spheroids, but not in loosely compacted aggregates. Additionally, a high level of CTGF expression was only observed in 3D-cultured HCCs, not in monolayer-cultured HCCs, even though Hep3B, Huh7, and SNU475 cells have fibroblast-like characteristics in monolayer culture (Figure 3A, 6A). CTGF is strongly induced by TGF-β and is tagged for secretion to the ECM under physiological conditions, whereas, overexpression of CTGF in tightly compacted HCC spheroids is induced regardless of expression of TGF-β1 (Figure 6A).

RT-PCR and real-time PCR analysis revealed that high expression of CTGF mRNA was similar among all monolayer-cultured HCC cell lines but differed between tightly compacted spheroids and loosely compacted aggregates in HCC, similar to the findings for COL1A1 mRNA expression (Figure 6B, 6C). We evaluated whether CTGF had an inhibitory effect on COL1A1 expression in tightly compacted spheroids and observed that CTGF knockdown significantly reduced COL1A1 synthesis (Figure 6D), decreasing interaction between HCC cells (Figure 6E) and resulting in greater therapeutic efficacy of anticancer drugs, which are sorafenib and cisplatin, in HCC spheroids (Figure 6F).

### Losartan attenuated the compactness of tumor spheroids and markedly improved efficacy of chemotherapeutic drugs in tumor spheroids

**Figure 7:**
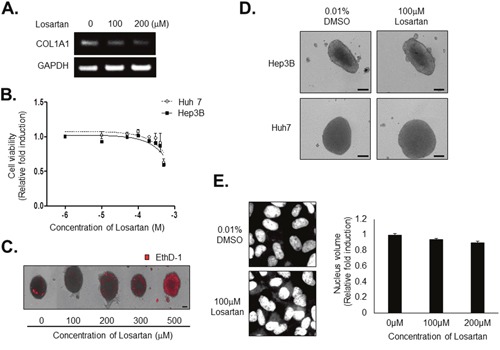
Losartan inhibits COL1A1 synthesis and attenuates the compactness of spheroids without cytotoxicity **A**. HCC spheroids were treated with 100 μM losartan for 3 days, and COL1A1 expression was evaluated by RT-PCR. GAPDH served as a loading control. **B**. Huh7 and Hep3B cells were treated with losartan at the indicated concentrations for 48hr. After treatment, nuclei were stained with Hoechst 33342 and counted. **C**. HCC spheroids were treated with losartan at the indicated concentrations for 3 days. Losartan-induced cell death was measured by staining with EthD-1. **D**. Huh7 and Hep3B spheroids were treated with 100 μM losartan for 3 days, and spheroid morphology was examined. **E**. Huh7 cells in monolayer culture were treated with 100 μM losartan for 72hr and nuclei were stained with Hoechst 33342. After image acquisition (left panel), nuclear volume was measured (right panel). Bright-field images were obtained using the same method with 10× objective for spheroids and 200× objective for monolayer cells. Data represent the mean values ± SD from three independent experiments relative to the value for control. Scale bar = 200μm.

We then investigated the mechanism underlying the effect of losartan on compactness of HCC spheroids. Losartan is widely used to treat hypertension as an angiotensin II type 1 receptor (AT1) antagonist with noted antifibrotic activity and, therefore, might decrease collagen I in *in vivo* tumors. We found that losartan inhibited COL1A1 mRNA expression in tumor spheroids (Figure 7A). Cell viability assays revealed that losartan did not have a significant cytotoxic effect on Huh7 or Hep3B cells (Figure 7B). EthD-1 staining to measure cytotoxicity of losartan in HCC spheroids showed that losartan induced severe cell death from a concentration of 300 μM treatment (Figure 7C). Interestingly, treatment with 100 μM losartan significantly increased the size of spheroids generated by Huh7 or Hep3B cells (Figure 7D) without changing nuclear or cell size (Figure 7E). These results show that losartan might regulate the interaction between the tumor cells without an anticancer effect in tumor spheroids.

**Figure 8:**
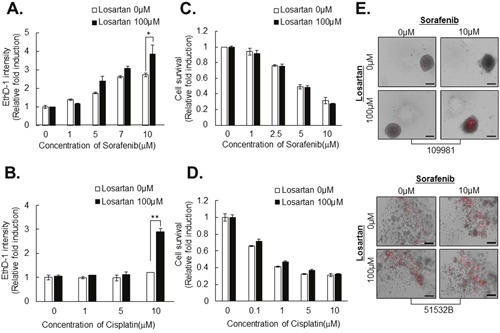
Losartan attenuates resistance to anticancer drugs in HCC cell line- and patient-derived primary HCC spheroids HCC spheroids were treated with 100 μM losartan for 3 days and then with sorafenib **A**. or cisplatin **B**. at the indicated concentrations for another 4 days. Then spheroids were stained with EthD-1 to detect cell death. HCCs in monolayer culture were treated with sorafenib **C**. or cisplatin **D**. at the indicated concentrations for 48 h, and nuclei were stained with Hoechst 33342 and counted. **E**. Patient-derived compacted spheroids (upper panel) and loosely compacted aggregates (lower panel) were treated with 100 μM losartan for 3 days and then with 10 μM sorafenib for another 4 days, and then death was measured by staining with EthD-1. All bright-field and fluorescence images were obtained using the same method. Data represent the mean values ± SD from three independent experiments relative to the value for control. *P<0.05. Scale bar = 200μm.

Next, we investigated whether structural changes in HCC spheroids induced by pretreatment with losartan overcame chemoresistance in tightly compacted HCC spheroids. Intensity of EthD-1 fluorescence and spheroid size were measured following treatment with anticancer drugs, sorafenib and cisplatin with or without pretreatment with losartan in Huh7 spheroids. Losartan pretreatment significantly enhanced sensitivity to anticancer drugs in a dose-dependent manner in Huh7 spheroids (Figure 8A, 8B), whereas pretreatment did not have this effect in monolayer cultures (Figure 8C, 8D).

We also investigated the effects of combination treatment with losartan and sorafenib in primary HCC tumors to provide better physiological relevance and applicability, and found pronounced therapeutic efficacy in tightly compacted spheroids when losartan was combined with sorafenib to treat human primary HCC cells (Figure 8E, Table 1).

**Table 1:** Combination effect between losartan and sorafenib in patient tissues-derived tumor spheroids

Patient sample	Type of Spheroid	Combination effect between Losartan and Sorafenib
51532B	Loose spheroid	=
15s-81449	Loose spheroid	=
AMC-H1	Loose spheroid	+
14s-103030	Loose spheroid	+
48551	Compact spheroid	+
15s-25590	Compact spheroid	+
103209	Compact spheroid	+
109981	Compact spheroid	+
101509	Compact spheroid	+
103850	Compact spheroid	+
AMC-H2	Compact spheroid	+

^*^Values of combination effect were normalized to single treatment of 5μM sorafenib. The + represented enhancement of anticancer effect, = represented nothing of significant change of anticancer effect by combination treatment 100μM losartan and 5μM sorafenib.

Taken together, our data suggest that treatment with losartan might attenuate the strong cell-cell adhesion by suppression of COL1A1 synthesis in tightly compacted HCC spheroids, thereby facilitating the robust therapeutic activity of combined losartan and anticancer therapies in human liver carcinomas.

## DISCUSSION

In this study, we highlight the importance of 3D cell-culture systems as a valuable methodology to understand mechanisms of chemoresistance by the TME in HCC and provide new insights into the biological functions of CTGF-induced collagen I expression in CAM-DR using the HCC spheroids.

We used the ultra-low attach (ULA) plate, which let the cells interact each other during their aggregation naturally without any treatment, for making the spheroids. We allowed HCC spheroids to form in culture and observed two groups, tightly compacted and loosely compacted aggregates with health condition (Figure 1, Supplementary Figure 1, and 2). We observed that 63.63% of human primary HCC cells formed tightly compacted spheroids (Table 1).

In practice, loosely compacted aggregates are not the optimal *in vitro* models because they lack diffusion barriers and have a stratified cellular composition, with proliferating cells at the surface and quiescent and necrotic cells in the core. On the other hand, tightly compacted spheroids more closely resemble the 3D cellular context, tumor complexity, and heterogeneity, as well as the therapeutically relevant pathophysiological gradients of *in vivo* tumors [14, 15].

Accordingly, growth in 3D culture has been shown to have dramatic effects on cell polarity and differentiation, as well as on signaling cascades and gene expression profiles, compared to growth in monolayer culture [16, 17]. In particular, liver cells preformed more liver–cell-specific functions in 3D versus 2D culture [18–20]. Therefore, 3D *in vitro* cell culture has been used in HCC research as a model intermediate between *in vitro* cancer cell line culture and *in vivo* tumor.

Here, we identified critical differences between tightly compacted and loosely compacted HCC aggregates in the presence or absence of fibroblast-like characteristics and drug resistance (Figures 2-4). Generally, 90% of HCC cases have a natural history of severe fibrosis (or cirrhosis) [21] and are resistant to conventional chemotherapeutic agents. Fibrolamellar HCC is characterized by laminated fibrous layers and does not respond to chemotherapy [22, 23]. Based on these clinical results, we speculated that the severely fibrotic HCC environment might contribute to evasion of anticancer therapies in HCC. When HCC progresses from fibrosis, the normal structure of liver is lost, with the formation of increasingly dense fibrous septae of fibronectin and collagens I, III, and V. In Figures 3 and 4, we revealed that COL1A1, which is regarded as the most important collagen in fibrosis, is upregulated in all tightly compacted spheroids. Moreover, COL1A1 knockdown improved the effect of therapeutics via structural changes that cells were dissociated from the center of spheroids and less compacted in the TME (Figure 5, Supplementary Figure 3). Hence, HCC cells with fibroblast-like characteristics facilitated rigid cell-cell adhesion and cell-COL1A1 interaction and thereby enhanced CAM-DR in tumor spheroids.

CAM-DR is rapidly induced by signaling events that are initiated by growth factors present in the TME. Here, we showed that overexpression of CTGF was correlated with tendency of COL1A1 expression in tightly compacted spheroids, and siRNA-mediated knockdown of CTGF efficiently reduced COL1A1 expression. Additionally, we found that CTGF knockdown resulted in greater therapeutic efficacy of anticancer drugs through induction of structural vulnerability in tightly compacted spheroids (Figure 6). CTGF can increase collagen in the human body [24–26] and has been investigated for its role in various fibrotic and desmoplastic diseases, including fibrotic skin disorder, organ fibrosis, and HCC [27–29]. Consequently, it is not surprising that CTGF expression is increased in tightly compacted spheroids. However, the most important point is that functional studies of CTGF should be conducted using the HCC spheroid models, because sufficient differences in CTGF expression between HCC cell lines can be detected in 3D, but not in monolayer, culture systems (Figure 6).

Generally, CTGF is transcriptionally activated by TGF-β, and enhanced expression of TGF-β has been demonstrated in monolayer-cultured HCC [30, 31]. Whereas, tightly compacted HCC spheroids exhibited TGF-β–independent CTGF induction (Figure 6A). Angiotensin II directly induced CTGF and collagen I expression through a TGF-β-independent Smad signaling pathway [32]. Losartan is an angiotensin II receptor antagonist used mainly to treat high blood pressure and potentiate chemotherapy through improvement of drug delivery, nanotherapeutics in tumor [32, 33]. Here, we determined the effect of losartan on Huh7 HCC spheroids and patient-derived primary HCC spheroids. An even more interesting fact is that losartan facilitates dissociation of strong cell-cell interactions and increases the therapeutic efficacy of conventional anticancer therapy in tightly compacted spheroids, not in the monolayer culture system (Figure 8, Table 1). To date, no single agent or combination therapy has been demonstrated to have any advantage in terms of both overall survival and quality of life, representing an unmet need. Combination therapy has not improved overall survival, but has nonetheless been in wide use for many years because of its possible roles in palliation. Thus, we herein suggest that combination therapy with losartan and existing anticancer therapies should be feasible for patients with HCC without imposing side effects, because losartan is already approved to treat hypertension without safety issues [34].

In conclusion, our results provide clear evidence that TGF-β–independent CTGF induction resulted in a rigid TME through increased COL1A1 expression and, thereby, plays a central role in CAM-DR in HCC. Inhibition of COL1A1 expression by treatment with losartan increased the therapeutic efficacy of anticancer therapies in HCC spheroids via disruption of CAM-DR. Therefore, our results suggest that a combination of losartan and conventional chemotherapy might be a promising approach to overcoming resistance to therapy in liver cancer.

## MATERIALS AND METHODS

### Cell lines and culture conditions

Huh7, Hep3B, PLC/PRF/5, SNU475, HepG2, and SNU449 human HCC cell lines were obtained from the Korean Cell Line Bank. Huh6 was kindly provided by Dr. Ralf Bartenschlager (University of Heidelberg, Germany). Cells were maintained at 37°C in a humidified atmosphere of 5% CO_2_. All HCC cell lines were cultured in Dulbecco's modified Eagle medium (DMEM; Welgene, Korea) supplemented with heat-inactivated 10% fetal bovine serum (FBS; Gibco, Grand Island, NY, USA) and 1× penicillin-streptomycin (Gibco) (Complete media).

### Primary culture of HCCs

Immediately after surgery, a portion of the tumor was immersed in Hanks balanced salt solution (HBSS; Gibco) and transported from the operating room at 0°C to the laboratory. The specimens were collected under sterile conditions and rinsed 2-3 times with HBSS free of calcium and magnesium to remove blood. After removal of blood, the liver sample was excised, cut into small fragments, gently dispersed and placed in HBSS containing 0.03% pronase, 0.05% type IV collagenase, and 0.01% deoxyribonuclease (DNase, from bovine pancreas) for 20 minutes at 37°C. The resultant was filtered through a 100 μm-nylon filter (BD Falcon, Franklin Lakes, NJ, USA) and centrifuged at 50x g for 2 minutes at 4°C to obtain hepatocytes. The pellet was washed twice in HBSS containing 0.005% DNase. The final cell suspensions were cultured onto collagen-coated T25 flasks (BD Falcon) in F12/DMEM (Gibco), supplemented with 20% FBS, 1% NEAA, 1% glutamine, and 1% P/S at 37°C in a humidified 5% CO_2_ incubator. The medium was changed 24 hours after seeding to remove dead cells and debris. When confluence reached 70-80%, the cells were re-plated using a 1:1 mixture of DMEM medium and F12/DMEM with supplements. After five passages, the cells were grown DMEM medium supplemented with 10% FBS and 1% P/S. Confluent cells were trypsinized, counted and split 1:3-1:5 at every passage. Once cell lines were maintained over 30 passages, they were collected and stored in liquid nitrogen.

### Generation of tumor spheroids and drug treatment

To generate spheroids, cells suspended in complete media were seeded at a density of 6×10^3^ cells/well in 96-well round-bottom ultra-low attachment (ULA) microplates (Corning B.V. Life Sciences, Amsterdam, The Netherlands). Plates were incubated for 3 days at 37°C in a humidified atmosphere of 5% CO_2_. To examine tumor-spheroid kinetics, images of cultured spheroids were obtained after 24hr later after plating and every 24hr after that. Spheroid diameter was measured using Operetta® Harmony software (PerkinElmer, Waltham, MA, USA). For drug treatment, cells were seeded with or without 100μM of losartan (Sigma) for 3 days and then with 1∼10μM of sorafenib (Santa Cruz Biotechnology, Santa Cruz, CA, USA) or cisplatin (Sigma) for another 4 days. Seven days after cell seeding, the spheroids were analyzed using the Operetta® High Content Screening System (PerkinElmer).

### Cell death detection in spheroids

Dead cell was detected using the cell-impermeant viability indicator ethidium homodimer-1 (EthD-1; Invitrogen, Eugene, OR, USA). EthD-1 is a high-affinity nucleic acid stain that fluoresces weakly until binding to DNA and emits red fluorescence (excitation/emission maxima ∼528/617). Spheroids were incubated in 4 μM EthD-1 in complete medium for 30 min in a 37°C incubator, and images were then obtained using a fluorescence microscope.

### High content screening and imaging technology

All bright-field and fluorescence images were obtained using the Operetta® High Content Screening System (Perkin Elmer) with a 10× objective for spheroids and a 40× objective for monolayers. The fluorescence images were captured according to the optimal excitation and emission wavelengths of each probe. To capture a sufficient number of cells (>100) for analysis, five image fields were collected from each well. Image analysis was performed using Hoechst 33342 (Invitrogen) staining of live cell nuclei and EthD-1 staining to detect cell death.

### Resazurin assay

Spheroids were generated as described above and treated with losartan and sorafenib. Spheroids were incubated in the presence of 50 μM resazurin (Sigma) for 5 h, and resazurin reduction was measured colorimetrically (570/600 nm) using the Enspire (Perkin Elmer) plate reader.

### Short interfering RNA (siRNA) transfection

siRNA probes were designed by and purchased from Bioneer (Daejeon, Korea), Huh7 and SNU475 cells were seeded and, when the cell density reached 40%–50%, medium was replaced with Opti-MEM (Gibco) without FBS and antibiotics. The sequences of si+COL1A1 and siCTGF were as follows: COL1A1 #sense, 5′-GUGCAUUCAACCUUACCAA-3′; COL1A1 #antisense, 5′-UUGGUAAGGUUGAAUGCAC-3′, CTGF #sense, 5′-GAAAAGAUUCCCACCCAU-3′; #antisense, 5′-AUUGGGUGGGAAUCUUUUC-3′. Cells were cotransfected with four siRNAs targeting COL1A1 (siCOL1A1), CTGF (siCTGF) and scramble (siCont) for 24 h using Lipofectamine 2000 (Invitrogen).

### Polyacrylamide gel electrophoresis (PAGE) and western blot analysis

Cells were solubilized in lysis buffer (3M, Maplewood, MN, USA), samples were boiled for 5 min, and equal amounts of protein (10∼30 μg/well) were separated on 10% or 12% SDS-PAGE gels. After electrophoresis, proteins were transferred onto a polyvinylidene difluoride (PVDF) membrane (Millipore, Billerica, MA, USA) and blocked with 5% of skim-milk or bovine serum albumin (Santa cruz) (for CTGF) for 30min at R.T. After blocking, PVDF membrane was incubated with anti-CTGF (Abcam, Cambridge, USA), anti-TGFβ1(Abcam), and β-actin (Sigma) for 16 hr at 4°C. After washing, blots were incubated with horseradish peroxidase-conjugated secondary antibody (Cell Signaling Technology, Danvers, MA, USA) diluted at 1:10000, and specific bands were visualized by chemiluminescence (ECL; Thermo Scientific, Waltham, MA, USA). Chemiluminescence was recorded onto X-Omat AR films (Eastman Kodak Co., Rochester, NY, USA).

### Reverse transcription-polymerase chain reaction (RT-PCR) and real-time polymerase chain reaction (real-time PCR)

Total RNA was isolated from cells using TRIzol® (Invitrogen, Eugene, OR, USA) according to the manufacturer's instructions. The reaction mixtures were comprised of RT buffer (Bio Basic, Amherst, NY, USA), dNTP solution (Bio Basic), RNasin® Inhibitor (Promega, Madison, WI, USA), oligo (dT)^15^ primer (Bioneer), total RNA, and M-MLV reverse transcriptase (Invitrogen). The reaction mixtures were incubated at 37°C for 1 h and the transcription reaction was terminated by heating the mixture to 95°C for 5 min and then rapidly cooling it on ice. The number of PCR cycles used was 30 for all reactions. The PCR products were then separated by 2% agarose gel electrophoresis and visualized with 5× Loading Star (Dynebio, Seoul, Korea). For real-time PCR, the mixture composed of cDNA, SYBR Green master mix (Applied Biosystems, Waltham, MA, USA), primers and DEPC, were performed using a StepOnePlus real-time PCR system (Applied Biosystems). The reactions were incubated in a 96-well optical plate at 95°C for 10 min, followed by 40 cycles of 95°C for 15s and 60° for 10 min. The threshold cycle (CT) is defined as the fractional cycle number at which the fluorescence passes the fixed threshold. CT values were normalized to GAPDH, and calculated according to the mathematical model R = 2−ΔΔCT, where Δ CT = CTtarget gene − CT_GAPDH_, and ΔΔ CT = ΔCTtest − ΔCTcontrol. All real-time PCR was performed in triplicates, and the data are presented as the mean ± SD. All primers were designed and purchased from Bioneer.

### Statistical analysis

All experiments were performed at least three times. The results are expressed as the mean ± standard deviation (SD). Statistical analysis was performed using Student's *t*-test.

## SUPPLEMENTARY MATERIALS FIGURES


