# Supplementary material for: TGF-β–independent CTGF induction regulates cell adhesion mediated drug resistance by increasing collagen I in HCC
Source: Oncotarget. 2017 Feb 20;8(13):21650–62. doi: 10.18632/oncotarget.15521 (PMC5400613; doi:10.18632/oncotarget.15521)
Supplement: Supplementary file 1 [file oncotarget-08-21650-s001.pdf]

TGF-β–independent CTGF induction regulates cell adhesion mediated drug resistance by increasing collagen I in HCC

SUPPLEMENTARY FIGURES

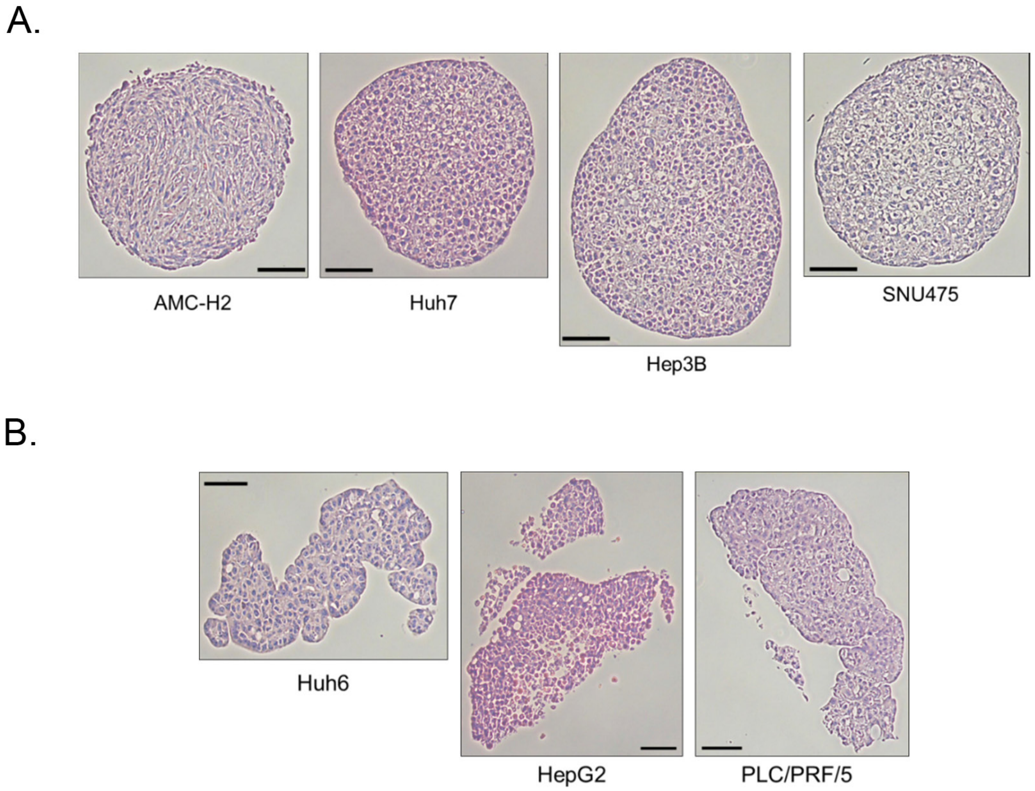

Supplementary Figure 1: H&E staining of tightly compacted spheroids A. and loosely compacted aggregates B. Scale bar = 100μm.

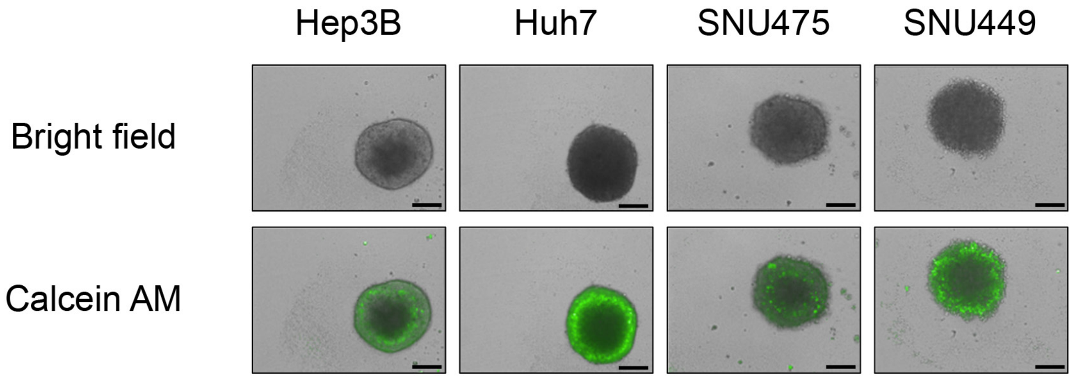

Supplementary Figure 2: Calcein AM staining of HCC spheroids for live cells. Scale bar = 200μm.

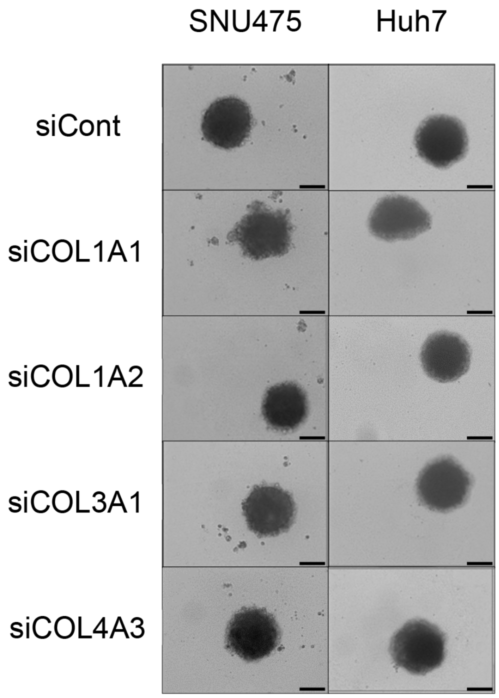

Supplementary Figure 3: Spheroid formation capacity of HCC cell lines, which were transfected with siRNA related to collagen. Scale bar = 200μm.
